# Association of Infants Small for Gestational Age with Anemia under Five Years Old in Two Large Longitudinal Chinese Birth Cohorts

**DOI:** 10.3390/nu14051006

**Published:** 2022-02-27

**Authors:** Nan Li, Hang An, Ming Jin, Zhiwen Li, Yali Zhang, Le Zhang, Jianmeng Liu, Rongwei Ye

**Affiliations:** 1 Ministry of Health Key Laboratory of Reproductive Health, Institute of Reproductive and Child Health, Peking University Health Science Center, Beijing 100191, China; linan01@pku.edu.cn (N.L.); anhang@bjmu.edu.cn (H.A.); 1610306233@bjmu.edu.cn (M.J.); lizw@bjmu.edu.cn (Z.L.); zhangyl@bjmu.edu.cn (Y.Z.); zhangle@bjmu.edu.cn (L.Z.); liujm@pku.edu.cn (J.L.); 2 Department of Epidemiology and Biostatistics, School of Public Health, Peking University Health Science Center, Beijing 100191, China

**Keywords:** small-for-gestational age, anemia, cohort study, infant, children

## Abstract

Babies who are born small for their gestational age (SGA) have low iron reserves, thus probably increasing the risk of offspring anemia. We studied two longitudinal birth cohorts to evaluate the association of SGA with the risk of anemia during early childhood. Cohort 1 was recruited from five counties in northern China involving 17,180 singleton infants born during 2006–2009 and cohort 2 from 21 counties or cities in southern China involving 180,619 children born during 1993–1996. Anemia was diagnosed by hemoglobin at 6 and 12 months in cohort 1 and at 55 months in cohort 2. The overall incidences of SGA were 7.07% and 5.73% in cohort 1 and cohort 2, respectively. SGA was associated with increased anemia at 6 months (adjusted odds ratio (OR): 1.52; 95% confidence interval (CI): 1.24, 1.86) and 12 months (adjusted OR: 1.42; 95% CI: 1.13, 1.79) in cohort 1 and at 55 months (adjusted OR: 1.11; 95% CI: 1.05, 1.17) in cohort 2. The positive associations for anemia at 6, 12, and 55 months persisted in both logistics and multiple linear models. Our results support a gradually decreased association between SGA and the increased risk of childhood anemia with a longer follow-up time in infants and children.

## 1. Introduction

Low birth weight, a surrogate marker of intrauterine malnutrition and developmental stressors, has emerged as a potential risk factor for neonatal, infant, and childhood health status [1,2]. The rates of LBW and infants who are small for their gestational age (SGA) have increased in recent years, and the current incidences in some Chinese regions are 5.2% [3] and 9.2% [4], respectively. Low-birth-weight infants delivered at term usually exhibit inadequate growth for their gestational age, whereas those delivered pre-term include those with normal growth for their gestational age and those with inadequate growth for their gestational age. Numerous research has focused on the association between anemia during pregnancy and SGA infants [5,6]. Little research has investigated whether SGA infants are associated with offspring anemia.

Anemia among infants and children is a global public health concern [7]. The hemoglobin concentration declines from a very high level at birth to its lowest level at about 2 months of age due to physiologic factors, then it increases again and becomes more or less stable at 6–9 months of age [8,9]. As its levels fluctuate with the ongoing life, an anemic status can occur at any stage of life, and it is essential to determine the likelihood of SGA infants being anemic at different young ages. Anemia is more prevalent in children aged < 5 years and especially in children aged < 2 years [10]. Infants [11,12] or children [13,14] with anemia could be fatigued or breathless in the short term, and this can lead to impaired behavioral and cognitive development in the long run. Research has found that 40–45% of children aged below five years suffer from anemia in developing countries [15]. As the largest developing country in the world, China plans to achieve their goal to reduce the anemia rate to <10% in children less than five years old. However, there is a large gap in our knowledge in China concerning the causes of childhood anemia at different ages.

Recent studies indicated children born with a low birth weight or SGA were positively associated with the risk of anemia, possibly due to their limited iron stores, which are proportional to body weight [9,16]. Insufficient iron intake may not meet the demands for red blood production, and thus the red blood cells will be abnormally small with low hemoglobin levels [17]. Therefore, we hypothesized that SGA is associated with increased risk of anemia at different follow-up time points under five years old and validated our hypothesis in two large-scale and well-characterized Chinese birth cohorts.

## 2. Methods

### 2.1. Background and Subjects for Current Study

Cohort 1 was derived from a randomized controlled trial that was implemented in five rural counties in the Hebei province of northern China. Briefly, 18,775 primiparous women were enrolled before 20 gestational weeks from 2006 to 2009 and were randomly assigned to three study groups (multiple micronutrients (MMN), folic acid plus iron (IFA), and folic acid (FA)), with the aim of evaluating whether prenatal supplementation with MMN or IFA versus FA could reduce perinatal mortality and other negative health outcomes for pregnant women and fetuses [18]. Of the 18,775 women from the northern province, we chose 17,748 women who registered on the monitoring system of offspring physical examination records as our target population. Of these women, we excluded 85 (0.48%) for whom the gestational age was unknown, <24 weeks or >43 weeks [19], along with eight (0.05%) with unknown or outlier infant birth weights, 43 (0.24%) with multifetal gestation, and 450 (2.54%) infants with no hemoglobin results at 5–7 months or 11–13 months. After these exclusions, 17,180 participants (96.80% of the targeted population) in cohort 1 were included in the final analysis.

**Figure 1:**
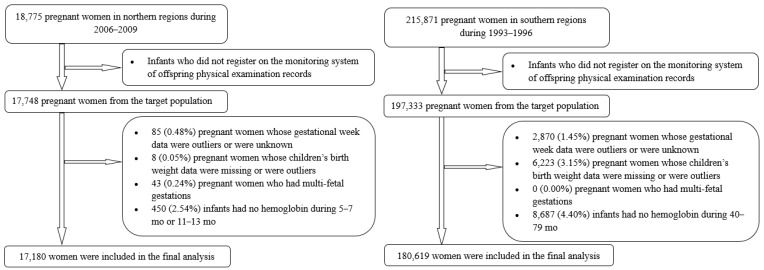
Flowchart of participants in cohort 1 (**left**) and cohort 2 (**right**).

Cohort 2 was derived from a large population-based prospective cohort study in 21 cities or counties in the Zhejiang and Jiangsu provinces of southern China. Briefly, 215,871 women in pregnancy during 1993–1996 were identified through the perinatal healthcare surveillance system and followed up between March and July 2000 for the measurement of anthropometric characteristics and hemoglobin at 40–79 months (mean: 55 months), with the aim to evaluate the effects of periconceptional use of 400 μg folic acid alone on neural tube defects, as well as child growth and development [20,21]. Of the 215,871 women from southern provinces, we chose 197,333 women who registered on the monitoring system of offspring physical examination records as our target population. Of these women, we excluded 2870 (1.45%) for whom the gestational age was unknown, <28 weeks or >45 weeks [22], 6223 (3.15%) with unknown or outlier infant birth weights, and 8687 (4.40%) infants with no hemoglobin results during 40–79 months. After these exclusions, 17,180 participants (91.53% of the targeted population) in cohort 2 were included in the final analysis. Information regarding formation of the target recruitment population and derivation of the population used in the final analysis is shown in Figure 1.

The original studies were approved by the Peking University Health Science Center Institutional Review Board. Secondary analyses of already collected data was deemed exempt from needing approval by the institutional review board.

### 2.2. Definition of SGA Status

Birth weight was measured in 10-g increments by trained local health workers within the first hour after delivery. To reduce inter-individual differences, a new, thin, clean cloth or piece of paper should be placed on the digital scale for each infant. The device is then adjusted to zero, the newborn placed on the scale naked, and the weight allowed to be stabilized before being captured and recorded. All instruments were well-calibrated by local authorities who ensured their quality and technical supervision.

Calculation of gestational age was based on the first day of the last menstrual period. Infants were considered SGA when the age-adjusted birth weight was <10th percentile based on results of a 2015 national survey [19] for cohort 1 and a 1998 national survey [22] for cohort 2.

### 2.3. Hemoglobin Measurement

In cohort 1, infants’ hemoglobin was measured by using capillary blood via the HemoCue system (HemoCue AB, Angelholm, Sweden). Before study initiation, township and county doctors were trained in standard measurement procedures. A step-by-step instruction leaflet was pasted on the wall of the township doctors’ rooms to ensure compliance with standard procedures. To minimize testing bias, the doctors’ rooms were equipped with heating devices in the winter to maintain a temperature of over 18 °C. In cohort 2, children’s hemoglobin was measured with a standard cyanmethemoglobin method by using capillary blood via devices available at each hospital; two commonly used devices were the 721 visible spectrophotometer and hemoglobinometer. All the project hospitals were provided with standard hemoglobin solutions (50, 100, 150, and 200 g/L) and with a step-by-step procedure for calibrating the hemoglobinometer and for preparing the standard curve for the 721 visible spectrophotometer. We defined anemia as hemoglobin concentrations < 110 g/L for infants and children aged < 60 months based on WHO recommendations [23].

### 2.4. Covariates’ Collection

All data for the two cohorts were obtained from perinatal healthcare surveillance systems via surveillance procedures and main questionnaire structures [18,24]. Surveillance systems in our county project areas were implemented through three-tier (county, township, and village) healthcare networks, whereas surveillance systems in city project areas were implemented through maternal and child health hospitals. Basic sociodemographic and prenatal healthcare records were first completed by experienced doctors in a brochure, then entered into computers by trained staff from the maternal and child health hospitals and transferred to the National Center for Maternal and Infant Health at the Peking University Health Science Center. Cohort 1 employed a network-based real-time data transfer system, whereas cohort 2 employed a telephone modem-based data transfer system. Project staff in Peking University Health Science Center were responsible for training the local health workers, data quality assurance, and annual report drafting.

The potential covariates for the two cohorts from surveillance systems included nutritional supplementation, maternal age, height, weight at first prenatal visit, hemoglobin levels during pregnancy, ethnicity, education, and occupation status.

### 2.5. Statistical Analyses

Continuous variables were calculated as the mean ± standard deviation (SD), and categorical variables were presented as the number and proportion. We used Student’s *t*-test and the chi-squared test to compare quantitative variables and categorical variables between newborns with or without SGA. Multiple logistic regression was performed to evaluate the odds ratios (ORs) of SGA on childhood anemia. We also calculated the mean differences to compare hemoglobin concentrations between SGA and non-SGA groups and to compare mean birth weights between anemia and non-anemia groups. All the regression models were adjusted for maternal age, BMI, education, occupation, ethnicity, anemia during pregnancy, feeding practices, and age at follow-up visit. Cohort 1 was also adjusted for micronutrient supplementation, while cohort 2 was additionally adjusted for parity and folic acid intake. Data were analyzed with SPSS version 20.0 software (SPSS Inc., Chicago, IL, USA). A two-sided *p*-value < 0.05 was treated as statistically significant.

## 3. Results

The maternal and child characteristics are shown in Table 1. Among the 17,180 children in cohort 1, 1214 (7.07%) were born with SGA status, whereas among the 180,619 children in cohort 2, 10,353 (5.73%) were born with SGA status. For both cohorts, children born with SGA status were more likely to have a mother of younger age and less body size. The mothers of the SGA group in cohort 2 were more likely to be of non-Han ethnicity. Most mothers were educated to junior high or higher school level in cohort 1, while the education level was more likely to be junior high or primary school in cohort 2. For both cohorts, mothers of SGA group were less educated. Over half of the mothers were farmers. There were no differences in how many mothers had anemia during pregnancy between the SGA group and non-SGA group. Most of the mothers were exclusively breastfeeding 42 days after birth. The mother of the SGA group was more likely to be non-exclusively breastfeeding in both cohorts. The mean age at follow-up for children in cohort 1 was similar across the two groups, whereas SGA children were older than those born with non-SGA status in cohort 2.

The overall anemia prevalence at 6 and 12 months in cohort 1 was 6.79% and 5.27%, respectively, and that at 55 months in cohort 2 was 13.21%. Children born with SGA had a significantly higher prevalence of anemia in both cohort 1 and cohort 2. In crude analyses, SGA children were associated with increased anemia at 6 months (OR: 1.55; 95% CI: 1.27, 1.89), 12 months (OR: 1.47; 95% CI: 1.17, 1.84), and 55 months (OR: 1.10; 95% CI: 1.04, 1.17). After adjustment for potential confounders, associations of SGA with anemia at 6, 12, and 55 months remained almost unchanged (ORs (95% CI): 1.52 (1.24, 1.86), 1.42 (1.13, 1.79), and 1.11 (1.05, 1.17), respectively) (Table 2).

The mean (±SD) hemoglobin concentrations at 6, 12 (cohort 1), and 55 months (cohort 2) were 121.71 ± 8.67 g/L, 122.07 ± 8.18 g/L, and 119.51 ± 10.19 g/L, respectively. The hemoglobin levels at 6, 12, and 55 months were −1.72 g/L (95% CI: −2.23, −1.22 g/L), −1.02 g/L (95% CI: −1.50, −0.54 g/L), and −0.21 g/L (95% CI: −0.41, −0.01 g/L) lower among SGA children relative to those of non-SGA children; the difference remained almost unchanged after adjustments for potential confounders (Table 3).

A higher likelihood of children’s anemia was observed among infants with SGA status in not only binomial (Table 3) but also multiple (Table 4) regression models. Table 4 shows that infants’ birth weights (mean ± SD) at 6 and 12 months in cohort 1 and at 55 months in cohort 2 were 3251.80 ± 441.10 g, 3257.92 ± 416.90 g, and 3291.08 ± 418.38 g for the anemic group, whereas those at the respective follow-up times were 3301.06 ± 380.68 g, 3299.93 ± 383.32 g, and 3303.00 ± 420.05 g for the normal group. We also found an obvious association between SGA and children’s hemoglobin at 6, 12, and 55 months using the birth weight as the main exposure variable (per 1-SD increase). Gradually weaker associations between SGA and childhood anemia were also observed with increasingly longer follow-up times in Table 2, Table 3 and Table 4, respectively.

## 4. Discussion

In these two large longitudinal Chinese birth cohorts, we found the SGA status to be associated with increased anemia in children aged 6, 12, and 55 months. The SGA–anemia association at 6, 12, and 55 months tended to persist across the infants’ different birth weights and hemoglobin levels. As far as we are concerned, this is the largest prospective longitudinal study to specifically examine the relationship between SGA status and childhood anemia. Our findings could provide great value in helping identify infants—specifically SGA infants—who may be at an increased risk of low hemoglobin levels in early childhood.

Previous studies have demonstrated that adverse pregnancy outcomes are risk factors associated with the development of offspring anemia. A low birth weight or SGA was related to hemoglobin and serum ferritin concentration at 6 months [25,26], 8 months [27], and 12 months [25,26] of age. The observation that LBW or SGA infants had a higher risk of lower hemoglobin levels most likely reflects newborns’ poor nutritional status. A study indicated that children born with very low birth weights were also more likely to have anemia (adjusted OR = 4.28, 95% CI: 2.67, 6.87) when aged 6–59 months in India [16]. However, the presence of anemia was computed based on population models. There might be differences between real individuals’ hemoglobin values and the predictive values from models. Data from China Nutrition and Health Surveillance indicated that LBW in western China was associated with a higher risk (OR = 1.99; 95% CI: 1.28, 3.09) of infantile anemia when aged 0–23 months [28]. The cross-sectional design of the study could not evaluate temporal relationships between SGA and the prevalence of anemia. We comprehensively integrated two large birth cohorts to prospectively investigate the effect of newborns’ SGA status on their childhood anemia, and our results were concordant with those findings. In the analyses of continuous hemoglobin (Table 3) and continuous birth weight (Table 4), moreover, we observed that a lower birth weight was consistently associated with reduced hemoglobin levels in infants at 6, 12, and 55 months, which also coincides with those previous findings. Meanwhile, it should be noted that mothers of SGA children were more likely to be younger, of less body size, less educated, and to have adopted non-exclusive breastfeeding. These may have an effect on children with anemia. However, after adjustment for these covariates, we found that the association between SGA and childhood anemia was still statistically significant

Few studies in the literature have compared the effects of the neonatal birth weight on the childhood anemic status at different ages. For newborn infants, iron stores and hemoglobin concentrations fluctuated with growth, and childhood anemia might occur at different ages under five years. Some studies indicated that iron reserves at birth could nearly cover the demands of growth in the first four to six months [29]. Therefore, we diagnosed childhood anemia at 6 months, 12 months, and 55 months to reflect the effect of SGA at different ages. The effect size in our study ranged from 1.52 (1.24, 1.86) at 6 months and 1.42 (1.13, 1.79) at 12 months, to 1.11 (1.05, 1.17) at 55 months. The decreasing significance of association at different ages suggests that, while there may be an impact of SGA on childhood anemia, the long-term effects may wane. The presence of the association strengthened the adverse pregnancy outcomes and increased the risk of infants’ anemia. The SGA-anemia association was identified as gradually weaker at 6, 12, and 55 months in children. One possible reason for this might be due to lifestyle amelioration after birth. Parents and caretakers of offspring diagnosed with SGA, however, likely pay greater attention to the children’s nutritional status for the first 1000 days of healthcare and thereafter, and such behavioral changes likely reduce the anemic outcomes. Improving maternal and reproductive health may thus play an important role in reducing the prevalence of children’s anemia. However, this is only conjecture; further research is needed.

Several possible mechanisms may account for our findings. Biological plausibility for the persistence of the adverse effect of SGA status on anemia is provided by existing knowledge that iron stores inherited from newborns are repeatedly used for erythropoiesis later in life. So, infants born with SGA status, compared with those born in normal health, probably have poorer nutritional status. They are most vulnerable to early iron depletion postnatally [30,31]. As iron stores of newborn infants are also proportional to body weight, SGA infants have relatively small iron stores. These iron losses are not compensated for postnatally without substantial dietary supplementation. SGA newborns face obvious repercussions in terms of their future iron status, and they may not have adequate stores until 4–6 months of life [29]. The finding that infants with SGA are more likely to have a lower hemoglobin level is most likely due to the newborns’ inability to meet the high nutritional demands of offspring growth. Nutritional and iron deficiencies are both causes of anemia [16].

Our present study had several strengths. First, it used a population-based prospective cohort design, and data were collected on both the exposure and outcome, thus minimizing the risk of selection and recall biases. Our analysis included cohort 1 in northern China and cohort 2 in southern China, and more than 90% of the children, with information on their birth weight and hemoglobin values, were included in our final analysis. We reached the same conclusion about SGA-anemia, with respect to the children from both cohorts, which makes our results reliable. Both these two studies used perinatal healthcare surveillance systems to record and follow-up participants’ information. The measurement of birth weight and the collection of information such as education and feeding practices were performed by trained health workers with similar procedures. Children aged 6–59 months with reported hemoglobin levels were included in this study, thus allowing us to compare the effects at different follow-up ages. Additionally, the two birth cohorts had dramatically different socioeconomic settings, which reinforces the validity of the notion of SGA-anemia. The sample size was large enough to detect both overall and subgroup effects. Detailed data from clinical records allowed us to examine associations among more subtypes of SGA and infants at different ages.

Yet, research limitations should be acknowledged when interpreting our findings. Our secondary analysis was derived from nutritional intervention studies, and there might be a potential effect of different set-ups of the two cohorts on the meaning of the results. However, we adjusted the nutritional factors in our model and found that the association between SGA and childhood anemia barely changed. Beyond this, infants’ hemoglobin values were measured with different devices in the two birth cohorts, and the measurement method differences across devices were not evaluated, which could potentially have led to the misclassification of anemic outcomes, resulting in an underestimation of risk. Moreover, the ferritin level and transferrin saturation values indicate a human’s different iron status; yet, we cannot say whether iron deficiency was supported by the ferritin level or transferrin saturation due to the lack of serum iron indicators. Finally, the participants in our study were Han (China’s predominant ethnic group), so our results may not be generalizable to other populations.

SGA ranges from 3% to 10% worldwide, and two-thirds of SGA cases occur in Asia. It is estimated that China has the fifth-highest number of SGA births in the world [32]. Both the United Nations Sustainable Development Goals (UN SDGs) and Millennium Development Goals (MDGs) highlight the need to reduce children’s mortality and improve their health. It has been reported that SGA could increase the risk of infant death and growth retardation [33]. Our study indicates the association of SGA with an increased risk of childhood anemia at different ages under five years. This finding may contribute data that are important with respect to SGA management and the prevention of anemia for children under five years old.

## 5. Conclusions

Together with other studies, these findings strongly advocate for the need for more prenatal healthcare for women, to improve birth outcomes, and to help combat childhood anemia. This will have long-term impacts on improving children’s overall health. SGA can be easily recognized in newborns and could be used as an indicator to administer interventions that promote the hemoglobin-associated nutrition of the child. This condition can be screened after birth to prevent low hemoglobin levels in children’s early lives.

## Figures and Tables

**Table 1:** Maternal and child characteristics according to SGA group.

Characteristics	Cohort 1				*p*	Cohort 2				*p*
	SGA Group (N = 1214)		Non-SGA Group (N = 15,966)			SGA Group (N = 10,353)		Non-SGA Group (N = 170,266)		
	*n*	%	*n*	%		*n*	%	*n*	%	
Mother										
Age (years, mean [SD])	22.96 (2.56)		23.41 (2.84)		<0.001	24.50 (3.54)		24.92 (3.65)		<0.001
Body mass index (kg/m^2^, mean [SD])	21.53 (2.69)		22.35 (2.86)		<0.001	20.11 (2.00)		20.49 (2.02)		<0.001
Han ethnic group	1201	98.93	15,774	98.80	0.684	10,253	99.24	169,012	99.42	0.020
Education					0.001					<0.001
High school or higher	205	16.89	2903	18.18		875	8.45	18,193	10.69	
Junior high school	974	80.23	12,823	80.31		6041	58.35	101,454	59.59	
Primary school or lower, or unknown	35	2.88	240	1.50		3437	33.20	50,619	29.73	
Farmer occupation	1112	91.60	14,517	90.92	0.430	6160	59.50	101,528	59.63	0.794
Anemia during pregnancy	89	7.33	986	6.18	0.094	6582	63.58	109,970	64.59	0.113
Exclusive breastfeeding	1141	94.00	15,277	95.68	0.006	8820	85.19	148,475	87.20	<0.001
Child										
Age at follow-up visit, months	6.25 (0.49) ^a^		6.26 (0.44) ^a^		0.785 ^a^	55.72 (8.31)		55.46 (8.19)		0.002
	12.25 (0.47) ^b^		12.27 (0.43) ^b^		0.124 ^b^					

SGA, small for their gestational age. SD, standard deviation. ^a^ First follow-up visit. ^b^ Second follow-up visit.

**Table 2:** Crude and adjusted ORs of anemia for gestational SGA group compared with non-SGA group.

	Children with Anemia					
	SGA Group		Non-SGA Group			
Mean Age at Follow-Up	*n*	%	*n*	%	Crude RR (95% CI)	Adjusted RR (95% CI) ^a^
Cohort 1						
6 months	119	8.87	1048	6.64	1.55 (1.27, 1.89)	1.52 (1.24, 1.86)
12 months	89	7.51	817	5.12	1.47 (1.17, 1.84)	1.42 (1.13, 1.79)
Cohort 2						
55 months	1481	14.31	22,377	13.14	1.10 (1.04, 1.17)	1.11 (1.05, 1.17)

OR, odds ratio; CI, confidence interval. ^a^ Common confounders adjusted for in the multiple logistic regression for both cohorts included maternal age, BMI, education, occupation, ethnicity, anemia during pregnancy, feeding practices, and age at follow-up visit. One additional confounder for cohort 1 was micronutrient supplementation; additional confounders for cohort 2 were parity and folic acid intake.

**Table 3:** Crude and adjusted mean difference in hemoglobin (g/L) for SGA group compared with non-SGA group.

	SGA Group	Non-SGA Group		
Mean Age at Follow-Up	Mean ± SD	Mean ± SD	Crude Mean Difference (95% CI)	Adjusted Mean Difference (95% CI) ^a^
Cohort 1				
6 months	120.12 ± 9.13	121.84 ± 8.62	−1.72 (−2.23, −1.22)	−1.61 (−2.11, −1.11)
12 months	121.12 ± 8.53	122.14 ± 8.15	−1.02 (−1.50, −0.54)	−0.92 (−1.40, −0.45)
Cohort 2				
55 months	119.31 ± 10.31	119.52 ± 10.18	−0.21 (−0.41, −0.01)	−0.26 (−0.46, −0.06)

OR, odds ratio; CI, confidence interval. ^a^ Common confounders adjusted for in the linear regression for both cohorts included maternal age, BMI, education, occupation, ethnicity, anemia during pregnancy, feeding practices, and age at follow-up visit. One additional confounder for cohort 1 was micronutrient supplementation; additional confounders for cohort 2 were parity and folic acid intake.

**Table 4:** Crude and adjusted mean difference in birth weight (g) for anemic group compared with non-anemic group.

	Anemic Group	Non-Anemic Group		
Mean Age at Follow-Up	Mean ± SD	Mean ± SD	Crude Mean Difference (95% CI)	Adjusted Mean Difference (95% CI) ^a^
Cohort 1				
6 months	3251.80 ± 441.10	3301.06 ± 380.68	−0.008 (−0.012, −0.004)	−0.009 (−0.013, −0.005)
12 months	3257.92 ± 416.90	3299.93 ± 383.32	−0.005 (−0.009, −0.002)	−0.006 (−0.009, −0.002)
Cohort 2				
55 months	3291.08 ± 418.38	3303.00 ± 420.05	−0.003 (−0.005, −0.002)	−0.004 (−0.006, −0.003)

OR, odds ratio; CI, confidence interval. ^a^ Common confounders adjusted for in the linear regression for both cohorts included maternal age, BMI, education, occupation, ethnicity, anemia during pregnancy, feeding practices, and age at follow-up visit. One additional confounder for cohort 1 was micronutrient supplementation; additional confounders for cohort 2 were parity and folic acid intake.

## Data Availability

The data are available in the main text, or can be obtained by contacting the corresponding author (Nan Li).
